# Medical Sociology

**Published:** 1911-01-14

**Authors:** 


The Hospital

A Journal of

The Medical Sciences and Hospital Administration.

New Series. No. 206, Vol. VIII. [No. 1278, Vol. XLIX.] SATURDAY, JANUARY 14, 1911.

### Medical Sociology.

Recently we published an announcement with regard to the founding of an American Society of Medical Sociology. The fact that such an association has been started on the other side of the Atlantic is a proof of the vital interest that is being felt over there in outstanding questions that affect the welfare of the community, an interest which is only gradually being awakened among us. And yet no one can deny that these problems are as important to us as a nation as they are to Americans. The preamble of the Society's circular letter is as applicable to our conditions, to our circumstances, as it is to circumstances and conditions in the United States. "Recognising the intimate relationship between disease and the social economic system under which we live, recognising that many diseases are caused directly by our social conditions, that the efficiency or inefficiency of our treatment often depends on the economic condition of the patient, that there are many problems deeply and vitally affecting the welfare of mankind which are left practically untouched by any existing medical society . . ." are the words with which this circular opens, and their truth is apparent at a glance.

When we peruse the list of subjects which the committee has framed for investigation and discussion we are again at once struck by the fact that they are matters which concern us as much as they do medical men elsewhere. The need of a special department of health, under a definite head; tuberculosis as an economic disease; the demonstrable relationship existing between the strain of modern life and the increase of insanity; the ætiological factors in the spread of malignant disease; the best—that is to say, the most humane and at the same time the most effective—means of dealing with prostitution; the best methods of preventing venereal infection; the consideration of the facts with regard to sexual abstinence; the relative influence of heredity and environment on the physical, mental, and moral characteristics of the offspring; the question of marriage and divorce; the regulation of conception, and the whole problem of the justifiability of such regulation; abortion and its medico-ethical aspects; alcohol and the drink problem; infant mortality; occupational and trade diseases; food adulteration; quackery and Christian science; and, finally, the relation between the public and the profession in all its aspects. These are some of the points with which such a society may concern itself, and we have no doubt that the establishment of the association in America will be productive of much practical good.

In England there are various bodies that, to some extent at least, are already working along the lines which the founders of this new organisation have laid down. We have a Sociological Society, a Eugenics Society, a Medico-Sociological section of the British Medical Association, a Society for the Study of Inebriety, a Medico-Psychological Society, and various others that have done, and are doing, excellent spade-work; but it is permissible to question whether these societies are not wasting valuable energy in doing pioneers' labour, each on its own account, without taking into consideration the immense benefits which are likely to result from co-operation. It is impossible for a medical man, be he ever so lightly worried by the cares of practice, to belong to every one of these various bodies and to be an active worker in each. Human life is too short, human strength too weak to make it possible for him to attend all their meetings and to take part in all their discussions. In the circumstances it may be asked why these associations do not combine in some way, and thereby add to their usefulness, their strength, and their influence. The example of the various medical societies in London in affiliating themselves with the Royal Society of Medicine and becoming component parts of that body should be a stimulus in that direction. No one can honestly assert that these independent bodies have lost prestige by sinking their individuality in the Royal Society; they have not even lost their independence, for the creation of the various sections has enabled those interested in particular subjects to maintain their freedom of choice with regard to attendances at meetings, while the moral influence of so strong a body as the Royal Society is incalculably greater than the combined influence of separate and independent entities working for a common object. We do not suggest that the Royal Society should attempt to gather into its fold the various bodies that now concern themselves with sociological problems, but we do suggest that these bodies should form themselves into a central society on the model of the Royal Society of Medicine, and by so doing conserve their energy, increase their prestige, and consolidate their interests.

The time is ripe for the founding of a really influential and authoritative organisation that can handle knotty problems of sociological importance. At present these questions are discussed in whispers, and the public gets an entirely false impression of their relative importance. Many of the most far-reaching questions of the day are sociological in their nature, and of such a character that they may confidently be asserted to be medico-sociological as well. To take one only, the question of compulsory military training is to some extent of this nature, and in how far it trenches on the domain of the medical man those who have read Dr. Newman's latest report to the Board of Education will readily realise. The problem of divorce is another of a similar kind, and it is easy enough to find others. On these questions the profession has a right to be heard. It has more: it has a right to expect that its opinions should be considered. There is no question here of medical priestcraft; no presumption of setting up a mysterious idol in an Isis temple and expecting the laity to bow submissively to it. The medical aspects of all these great problems will bear discussion. Our opinion on them is formed on facts regarding which the public, as a whole, is wholly ignorant, and it is a moral obligation on us to pronounce that opinion and to support it by stating reasons, chapter and verse, for the conclusions. Such a society as we have suggested will be in an admirable position to act as a professional mouthpiece, and its influence will be materially more robust if it can number among its members lay as well as medical men.

